# The patient safety in extracorporeal blood purification treatments of critical patients

**DOI:** 10.3389/fneph.2022.871480

**Published:** 2022-07-22

**Authors:** Federico Nalesso, Francesco Garzotto, Tiziano Martello, Cristina Contessa, Leda Cattarin, Mariapaola Protti, Valentina Di Vico, Lucia Federica Stefanelli, Giuseppe Scaparrotta, Lorenzo A. Calò

**Affiliations:** ^1^ Nephrology, Dialysis and Transplant Unit, Department of Medicine, University of Padua, Padua, Italy; ^2^ Department of Cardiac Thoracic Vascular Sciences and Public Health, Unit of Biostatistics, Epidemiology and Public Health, University of Padova, Padova, Italy; ^3^ Department of Directional Hospital Management, Medical Directorate, Padua University Hospital, Padua, Italy

**Keywords:** patient safety, extracorporeal blood purification, critical care nephrology, acute kidney injury, risk management, chronic kidney disease

## Abstract

Today, health systems are complex due to both the technological development in diagnostic and therapeutic procedures and the complexity of the patients that are increasingly older with several comorbidities. In any care setting, latent, organizational, and systematic errors can occur causing critical incident harmful for patients. Management of patients with acute kidney injury (AKI) requires a multidisciplinary approach for the diagnostic-therapeutic-rehabilitative path that can also require an extracorporeal blood purification treatment (EBPT). The complexity of these patients and EBPT require a clinical risk analysis and the introduction of protocols, procedures, operating instructions, and checklists to reduce clinical risk through promotion of the safety culture for all care providers. Caregivers must acquire a series of tools to evaluate the clinical risk in their reality to prevent incidents and customize patient safety in a proactive and reactive way. Established procedures that are made more needed by the COVID-19 pandemic can help to better manage patients in critical care area with intrinsic higher clinical risk. This review analyzes the communication and organizational aspects that need to be taken into consideration in the management of EBPT in a critical care setting by providing tools that can be used to reduce the clinical risk. This review is mostly addressed to all the caregivers involved in the EBPT in Critical Care Nephrology and in the Intensive Care Units.

## Introduction

The term “patient safety” identifies the set of specific actions and tools aimed at preventing errors and their effects in a diagnostic-therapeutic process to which the patient may be exposed within the health system (1, 2).

Considering the complexity of the treatments that are currently provided in critical care settings [Intensive Care Unit (ICU), COVID-19 Unit, and Heart Surgery, Transplantation Unit], it appears clear that, as the complexity of healthcare increases, the possibility of medical errors occurs with potential clinical incident for patients (3).

The extensive use of new technologies, drugs, medical treatments, and multidisciplinary cares leads to increasing complexity of the healthcare system, requiring a structured communication between care providers. This is fundamental to ensure a correct information exchange within the diagnostic and therapeutic paths, particularly when the treatment of increasingly older and critically ill patients with comorbidities that often require difficult clinical decisions for their management is considered. The increase in economic pressure and assistance requests on the health system further leads to stressful and potentially unsafe working environments with work overloads, as often happened during the COVID-19 pandemic (4).

To achieve an efficient and quality health system, the patient safety becomes a constitutive element of the care process as the quality of care cannot exist without patient safety (5).

Going to a more analytical view of the risk management, we understand how adverse events can arise from organizational problems, problems associated with clinical practice, communication, drugs administration, patient’s hand-off, medical procedures, protocols, operating instructions, and use of medical devices (3, 6). Of particular importance is the periodic and systematic assessment of the care provided and reported incidents to better identify active and/or latent failure, to introduce corrective measures on organization’s processes. Medical errors can lead to different types of clinical incidents: in the “near miss”, the incident does not affect the patient, and in the “no harm incident”, the incident affects the patient without causing harm, whereas the “harm incident” affects the patient with a clinical harm (3).

In Critical Care Nephrology, the extracorporeal blood purification treatments (EBPTs) are source of clinical risk, due to their complexity and need for multidisciplinary approach in critical patients (7). Therefore, they become a high-risk procedure when not performed in a qualified setting where the staff expertise can determine the significant reduction in the standard risk (7).

The ICU is particularly prone to medical errors and adverse events resulting from the complexity of the environment where medication errors, injuries associated with airways or ventilator use, invasive-line complications, infections, and rapid deterioration in patients’ conditions are common elements to be considered. In addition, omission of therapies that should be administered accounts for significant and preventable morbidity, mortality, and costs associated with ICU care. In this setting, during EBPTs, errors may occur regarding the patient’s incorrect weight loss, the administration of systemic or loco-regional anticoagulation, unexpected hypovolemia that can lead to hypotension, and dysionemia due to incorrect fluids for hemodiafiltration.

Data reported by the first studies about patient safety performed in the USA showed that about 1 in 10 people receiving medical treatment develops preventable harm (8), therefore demonstrating the importance of the patient safety policy and justifying its extensive and systematic implementation in the clinical practice in all medical specialties. According to the largest meta-analysis assessing preventable medication harm, around 1 over 30 patients is exposed to preventable medication harm in medical care, and more than a quarter of this harm is considered severe or life-threatening (6). These results support the World Health Organization’s pressure for the detection and mitigation of medication-related harm as being a top priority while highlighting other key potential targets for remedial intervention that should be a priority focus for future research.

To ensure that medical incidents can be effectively prevented, the culture of patient safety must be widely promoted in every health system (9) by customizing protocols, procedures, operating instructions, communication, and the information technology introduction (10, 11) on individual local needs, avoiding standardized and inflexible procedures that result in ineffectiveness and unprotectiveness in dynamic and complex care settings.

This review analyzes the communication and organizational aspects that need to be taken into consideration in the management of EBPT in a critical care setting by providing general tools that can be used to reduce the clinical risk. This review is mostly addressed to all the caregivers involved in the EBPT in Critical Care Nephrology and in the ICUs.

## The patient safety in EBPT of critical patients

In critical patients, EBPTs have an important role not only in the replacement of renal function *via* control of acid–base, electrolyte, and fluid balance but also in the removal of specific pathogenic molecules such as endotoxin, myoglobin, cytokines, inflammatory factors, hepatic toxins, toxics, drugs, poisons, and carbon dioxide in the so-called decapneization treatment (12, 13). Because of the technological development, nowadays, we have many EBPTs that share these treatment indications, making the critical patient’s care complex, requiring a multidisciplinary approach. The more complex and multidisciplinary this approach, the greater are the chances that the clinical risk increases with the possibility of clinically relevant incidents.

To provide blood purification, specific hemodiafiltration solutions (14) and/or medical devices (filters with different membrane characteristics, adsorbent cartridges, and decapneization devices) are necessary in extracorporeal circulation managed by different software and hardware. The possibility of using this technology for the Extracorporeal Organ Support (ECOS) (15) and the Multiple Organ Support Therapy (MOST) (16) makes complexity even higher, requiring a multidisciplinary approach and specialized personnel.

It is evident that EBPT complexity is source of preventable and predictable incidents due to errors with a high burden of clinical risk, leading to different severity degrees of injury for patients.

The clinical pathway from the identification of EBPT indications and its prescription, administration, and management are complex and consist of many interconnected phases where an error can have repercussions in subsequent phases (7). In this process, the care provider who materially “makes the error” is not always the one who generated it. Hence, there is the need for tracing all the process phases and all the operators involved.

In detail, in EBPT in the critically ill patient as the error can occur in any phase with possible propagation, all phases of the EBPT process and their interconnections have to be identified and described in detail to act proactively with appropriate tools for error prevention.

Specifically, errors in the *parameters of the EBPT* prescription relate to incorrect nomenclature (17, 18), blood flow, dialysate flow, total reinfusion flow, unit of measurement of flows, pre- and post-dilution reinfusion percentage, filtration fraction (FF), inappropriate weight loss (19), anticoagulation settings (citrate, heparin, and low–molecular weight heparin dose) composition of hemodiafiltration bags, and, finally, the discrepancy between prescribed and administered dose that depends on real patient’s weight, dilution factor, and treatment downtime (**Table 1**). The use of *incorrect materials* relates to the wrong association between the blood purification technique and the filter characteristics depending on its intended use (20), and between patient’s acid–base and electrolytic balance and the hemodiafiltration fluid composition (14). These errors lead to an incorrect formulation of the treatment with possible iatrogenic damage to the patient (for example, electrolytes alterations). The most common errors that can be made by care providers are incidents related to the replacement of a type hemodiafiltration fluid with respect to the prescription, the reduction of blood flow due to repetitive alarms for the vascular access with reduction of the depurative dose, and the delayed maintenance of the alarms monitor with increased treatment downtime. All these elements can be detected at the periodic (daily) check of the EBPT by the specialized Nephrology staff who are responsible for verifying the consistency of the treatment with the prescription. It is suggested that this procedure should be checked according to a protocol and a coded checklist for each type of available treatment in the specific setting of care. We suggest that the most critical elements (weight loss, fluid balance, anticoagulation, blood flow, state of the extracorporeal circuit, central venous catheter, treatment parameters, and type of bags) have to be checked every 6-h intervals using a checklist that obliges care providers to verify each element. This process has to be shared among the different care providers in the multidisciplinary team for which correct communication becomes an essential point.

**Table 1:** Common types of errors, consequences, and possible corrective actions.

Errors	Consequences	Corrective Action
Errors in the nomenclature of treatment (E.g.: prescription of CVVHDF without specification of dyalisate flow rate and prescription of a reinfusion flow for a CVVH treatment without the pre-dilution percentage).	Mismatch between the prescription and the parameters set in the prescription. Possible misinterpretation of missing data or failure to enter data by nurses.	**Introduction of appropriate nomenclature and standardized prescription criteria for each treatment in an electronic or paper format**. The personalization of the treatment is guided by the standard prescription for a patient weight of 70 kg.
Errors in the prescription and incorrect choice of materials/devices that do not match to the type of treatment: • Types of hemodiafiltration solutions • Filters - plasma separators – Adsorber - Cartridge • Lines for extracorporeal circulation • Monitors (consider also software version and its updates for the use of special devices)	Mismatch of materials that cannot be used correctly for the selected depurative treatment. (E.g.: concentration of potassium inappropriate for the patient’s clinical condition, filter/device inappropriate for the treatment prescribed, and prescription of an ineffective depurative treatment for wrong or missing data)	**Standardization of each treatment** in terms of: ● Equipment ● Types of filters ● Types of lines for the extracorporeal circulation ● Types of hemodiafiltration solutions ● Settings of treatment ● Anticoagulation requirements and types of anticoagulation needed ● Storage of materials for easy availability with clear labels to associate the materials for each treatment Introduction of appropriate checklists to confirm the correctness of the association of materials, the preparation of the monitor for the treatment and the parameters setting required by the purification treatment
Errors in the settings of prescribed treatment: ● Blood flow rate (ml/min) ● Dialysate flow rate (ml/h or L/h) ● Reinfusion flow rate (ml/or L/h) ● Percentage of pre- and post-dilution in convective treatment (CVVH, CVVHDF) ● Prescription dose (ml/kg/h according to K-DIGO 2012). Consider influencing factors: ● Blood flow ● Filter type and surface ● Dialysate and reinfusion volume ● Dilution Factor due to the pre-dilution ● Filtration Fraction ● Total Downtime ● Partial Filter/Device clotting during treatment ● Maximum weight loss per hour (ml/h) ● Total weight loss (ml/day or L/day) ● Total time (hours and minutes) ● Heparin infusion rate (UI/h or UI/kg/h) ● Citrate and calcium infusion rate in RCA (Citrate in mmol/L of blood treated and Calcium compensation in mmol/h)	Mismatch between parameters required for treatment and parameters actually prescribed. Free interpretation of missing parameters in the prescription. Hourly weight loss and total weight loss per day not corrected with errors in the required fluid balance. Errors in the dose of purification. Appearance of alterations in the electrolyte and acid–base balance. Excessive or unwanted removal of useful substances such as antibiotics. Exposure to patient’s hemorrhagic risk or coagulation of extracorporeal circulation in case of use of heparin or LMWH. Alterations of calcium (hypercalcemia - hypocalcemia) and acid–base balance (alkalosis or acidosis) due to incorrect use of citrate up to citrate intoxication due to its altered metabolism.	**Introduction of appropriate nomenclature and standardized prescription criteria for each treatment in an electronic or paper format**. Introduction of **operating instructions** for prescribing treatments, priming the monitor, calculating the depurative dose (ml/kg/h) and its correction based on the expected downtime and the dilution factor due to pre-dilution. Continuous **staff training and retraining** also for treatments rarely performed. **Introduction of customized checklists** for the control of all critical and transition points between one phase and another of EBPT (prescription, monitor assembly, monitor priming, parameters setting, patient connection and disconnection, treatment supervision, and detection of treatment abnormalities). Design of **surveillance protocols** of RCA treatments for the verification and correction of calcium compensation and to promptly identify the accumulation of citrate or electrolyte and acid–base balance alterations. Introduction in the clinical practice of universally shared protocols for the control of ionemia, acid–base balance, depurative dose and fluid balance in each patient; it is useful to understand remote monitoring.
Types of anticoagulation: ● None ● Heparin ● LMWH ● Citrate	Risk of circuit clotting if heparin is not prescribed in patients with hemorrhagic risk. Increased risk of hemorrhage when heparin or LMWH anticoagulation are used in patients at risk. Alterations of calcium (hypercalcemia - hypocalcemia) and acid–base balance (alkalosis or acidosis) due to incorrect use of citrate up to citrate intoxication due to its altered metabolism in patient with liver failure or drug use interfering with mitochondrial metabolism.	Introduction of **circuit management procedures in the absence of anticoagulants** to avoid circuit clotting (increase in the percentage of predilution, reduction of the filtration fraction, use of diffusion only, increase in blood flow, and foresee the change of the circuit at fixed times). Introduction of **procedures for prescribing anticoagulants** in terms of dose of anticoagulants (heparin and LMWH) and monitoring of the effect on coagulation (ACT and aPTT). Introduction of **procedures for the preparation of heparin solutions in terms of concentration and infusion rate**. Introduction of **procedures for the management of patients on regional citrate anticoagulation with particular attention to the different types of fluids used**.

CVVHDF, Continuous Veno-Venous Henmodiafiltration; CVVH, Continuous Veno-Venous Hemofiltration; RCA, Regonal Citrate Anticoagulation; LMWH, Low–Molecular Weight Heparin; ACT, activated coagulation time; aPTT, activated partial thromboplastin time.

In fact, communication among care providers of critically ill patients is a constitutive element of the EBPT management. If not structured and codified, then the communication between the prescriber physician and nurse can be source of errors for the presence of incorrect or missing parameters that are consequently interpreted by the nurse during the treatment administration. To ensure the maximum level of safety, this communication process has to be codified through the use standardized forms, standardized protocols, procedures, and checklist tailored to the specific local setting that has to be previously mapped for clinical risks by a proactive analysis. This process can better identify active and/or latent failure, proposing corrective measures on organization’s processes. The communication modality and the structured tools must be widely known by all care providers to ensure their correct penetration at all levels of care (21).

The most critical point for communication is the handover (22, 23) that has to be structured to improve the safety by building a care setting in which information is encoded so that “the sender” and “the receiver” can understand whether the process has been correctly performed (21, 24). It is therefore useful to implement “the closed loop communication” (25) to make all the caregivers able to verify the exact and complete communication of the patient’s clinical condition by a clear and shared language in terms of parameters and nomenclature understandable by all the caregivers involved in the patient care. There are many models in the literature that can help caregiver to carry out the handover: One example is the SBAR model (Situation, Background, Assessment, and Recommendations) (26–28).

The EBPT management is complex and composed of several interconnected phases. The understanding of the treatment prescription by the nurse takes the form of preparing the monitor with the correct materials and setting the correctly parameters at the software level. Any error in reading or understanding the prescription, or the use of wrong materials or incorrect settings, generates an error that can result in the patient’s injury in the following phases. The use of checklists and operating instructions can reduce the clinical risk (29, 30) by introducing tools that block the progression of the treatment in the presence of errors or inconsistencies with respect to the treatment prescription. The extensive introduction of protocols and operating instructions, which describe in detail materials and modality to carry out each phase of the EBPT, determines a process standardization that can be controlled by all care providers in the identified critical points by checklists (31).

Team training plays a key role in increasing patient safety (32). It is necessary to provide continuous theoretical and practical training for personnel involved in the EBPT to make communication effective and to acquire the skills for patient management to prevent and promptly identify situations of potential risk. All caregivers need to be aware that they are part of a system that ensures safety by taking a proactive and reactive role in the clinical risk management by the process of incident reporting. Briefings and de-briefings should be supported to promote the “no blame culture” so that everyone will be encouraged to report an incident knowing that the focus is on “how and why” an incident happened rather than “who” made the mistake (33). It is known that the reactive approach based only on measures taken following the identification of an accident exposes patients to a clinical risk higher than a system based on a proactive and preventive method. As a proactive approach, it is recommended to perform the risk mapping by using FMEA (Failure Mode and Effect Analysis) and FMECA (Failure Mode, Effects and Critically Analysis) and to take a “safety walk round” (SWR) at least every 6 months (34). In case of incident, it is advisable to proceed with the Root Cause Analysis (RCA) and the systematic review of the safety tools introduced in clinical practice (35).

The patient safety in EBPT is complex and involves human, environmental, technical, and technological factors over which the complete control becomes a challenge, especially in the most critical care situations with work overload.

In many ICU settings, the treatment is prescribed by nephrologist and subsequently set and delivered by a trained nurse. Given the severity of patients in ICU with possible deterioration of hemodynamics, the treatments that are most prescribed are continuous [Continuous Kidney Replacement Therapy (CKRT)], even if in a minority of patients, it is possible to administer hybrid treatments [Sustained Low-Efficiency Dialysis (SLED)] or intermittent dialysis (IHD). For all these techniques, the clinical risk is present regardless of their duration of treatment over time and all treatments must be subject to the same safety monitoring. Therefore, the treatment supervision for CKRT and SLED is entrusted to ICU nurses who create a therapeutic alliance with nephrologists based on correct communication and adequate EBPT knowledge. These elements must be considered and verified to build a customized safety environment with tools for the clinical risk (7).

## Patient safety in performing EBPT during COVID-19

Patients with COVID-19 are generally admitted to ICUs for respiratory failure that can evolve into a multiorgan dysfunction syndrome requiring ECOS by sequential extracorporeal therapies designed to remove inflammatory mediators and support different organ systems (36) by hemoadsorption, hemoperfusion, and extracorporeal CO_2_ removal (ECCO_2_R) (37). In this setting, EBPT could be an option to prevent organ failure and improve survival at the cost of greater complexity, clinical risk, and demand for the availability of medical devices.

To predict the spread of coronavirus disease globally and consequently prepare the hospital facilities with the required technology is a challenge (38). The availability of essential medical equipment to support patients affected by COVID-19 is globally limited, and, in this exceptional situation, it is necessary to re-assess the risk analysis on medical equipment management and their use and re-use in this context with the aim to improve global healthcare also in term of devices for EBPT. Every effort must be made to provide the necessary devices at least with the minimum acceptable performances for patients with COVID-19 while maintaining a high standard of safety (39).

Patient safety is of particular importance during the COVID-19 pandemic (40), where there is an overload of the world health systems in a situation of lack of human resources and medical supply (38, 41, 42).

Care providers are predisposed to a greater propensity to error due to this sudden increased workload, the need to provide EBPT in environments other than the usuals, in a more complex care setting of untrained personnel for the Critical Care Nephrology due to the high turnover of medical teams.

The initial lack of protocols for the patients with COVID-19, especially in the field of EBPT, resulted in the lack of standardized management influenced by local resources and individual capacities to react to an unpredictable pandemic and therefore to the need of customized protocols and procedures for this patient population (43–45). The use of devices in an emergency setting out of its intended use or differently from the manufacturer’s instructions based on a specific risk (46) requires multidisciplinary evaluation aimed to re-assess risks and benefits (47).

All Kidney Replacement Therapy (KRT), and specifically EBPT, required a tailoring not only of anticoagulation and blood flow (48) but also of applicability in a population of critically ill patients with special care needs including biological risk for the care providers and the need for cytokines and endotoxin removal by specific devices to integrate into the extracorporeal circulation of KRT or in more complex system such as the Extracorporeal Membrane Oxygenation (ECMO).

The clinical risk in this pandemic situation is increased due to the possibility of delays in the management of extracorporeal circulation due to the biohazard, the increased extracorporeal circulation clotting due to the patient’s spontaneous hypercoagulability associated with the use of convection with a high FF with reduced use of regional citrate anticoagulation (49), and the difficult supervision of EBPT monitor, blood chemistry tests, and fluid balance often performed remotely due to the biohazard. Furthermore, blood losses for circuit clotting, errors in patient weights, and depurative dose and incorrect fluid balance increase the clinical risk with a reduction in the quality of the EBPT administered.

During the COVID-19 pandemic, all these clinical elements added to the sudden need for KRT in the overload health system without a designed plan for an appropriate response where the demand for dialysis services dramatically increased. The need for an adequate prediction of all aspects concerning medical personnel, disposable devices, and KRT monitors is nowadays fundamental for a response to this pandemic in compliance with patient safety (50).

## Discussion

In critical setting, the EBPT administration, due to its complexity and the patient’s critical illness, is a process that presents an intrinsic degree of “unsafety”. This was even more evident during the COVID-19 pandemic where no healthcare system was ready to face an emergency in terms of human resources, supply, and clinical risk management.

As the EBPT can be administered in a setting outside the Nephrology-Dialysis Unit with trained but unskilled staff, patients may be exposed to a greater clinical risk that can lead to errors and incidents.

**Figure 1:**
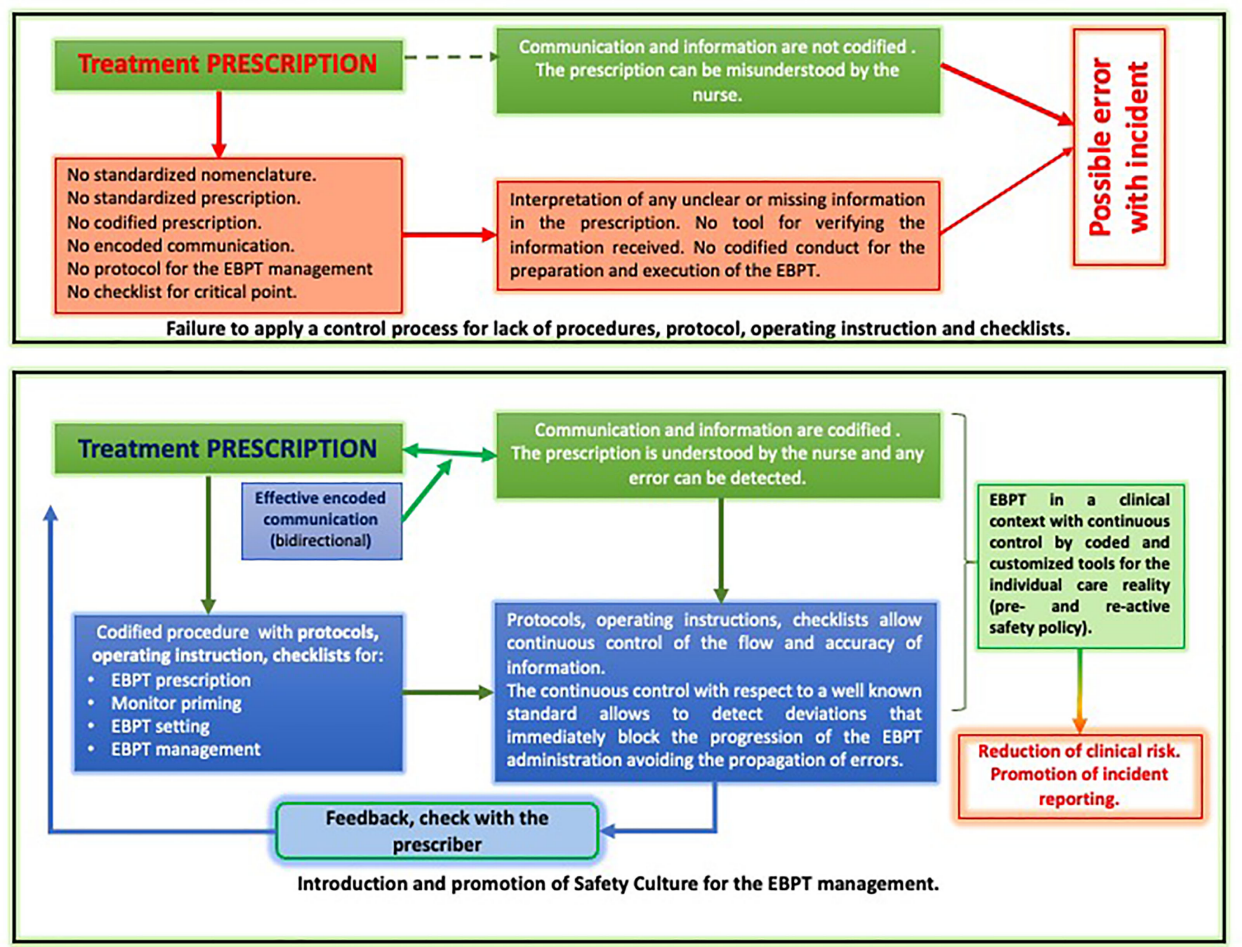
Patient safety integration in the EBPT management.

All these elements have to be taken into account to build a safe local reality based on the introduction of protocols, procedures, operating instructions, and checklists aimed at the clinical risk mitigation (**Figure 1**). These tools have to be created in relation to the proactive analysis and continuously implemented in a reactive approach based on the patient safety culture and incident reporting.

The coding of materials, devices, and phases of EBPT through protocols and the control of critical phases by checklists introduces essential tools for the control of clinical risk, inducing all care providers to comply with the safety standards introduced in the care setting.

The need of a standard nomenclature universally accepted and shared by all care providers determines a more effective and safe communication based on encodings, allowing the sender and the receiver to have prompt feedback on the quality and type of exchanged information.

The promotion of briefing, de-briefing, staff training, simulation of stressful situations, and work overload provides further tools for patient safety.

The extensive and continuous promotion of the culture of patient safety and incident reporting makes it possible to identify any potential risk or the occurrence of an incident that can be analyzed through tools (FMEA, FMECA, SWR, and RCA) for corrective measures.

Building a dynamic and non-static safety system makes it possible to deal quickly and safely with actual problems, as from the lesson of COVID-19 pandemic.

In conclusion, the patient safety and its specific actions and tools for preventing errors are the basis to build an efficient and quality health system where the patient safety becomes a constitutive element of the care process, as the quality of care cannot exist without patient safety. EBPTs have an important clinical role in critical patients not only in the replacement of renal function but also in the removal of specific pathogenic molecules in the ECOS and MOST that make their complexity even greater requiring an extensive clinical risk management. The clinical setting analysis for risks, the EBPT coding, and the introduction of protocols, procedures, operating instructions, checklists, models of communication among care providers, the promotion of the incident reporting, and the culture of safety, scheduling training, and retraining of all personnel make possible to manage EBPTs safer especially in conditions of clinical stress, workload, and biohazard, as occurred during the COVID-19 pandemic.

## Author Contributions

FN: concept/design, data interpretation, drafting article, critical revision of article, and approval of article. FG: drafting article and critical revision of article. TM: drafting the paper. CC: drafting the paper. LC: drafting the paper. MP: drafting the paper. VdV: drafting the paper. LFS: drafting the paper. GS: critical revision of article. LC: critical revision of article and approval of article. All authors contributed to the article and approved the submitted version.

## Funding

This study was supported in part by the grant DOR2114705 from University of Padua to FN.

## Conflict of Interest

The authors declare that the research was conducted in the absence of any commercial or financial relationships that could be construed as a potential conflict of interest.

The reviewer SR declared a past co-authorship with the authors FN to the handling editor.

## Publisher’s Note

All claims expressed in this article are solely those of the authors and do not necessarily represent those of their affiliated organizations, or those of the publisher, the editors and the reviewers. Any product that may be evaluated in this article, or claim that may be made by its manufacturer, is not guaranteed or endorsed by the publisher.

## References

[1] Kohn LT Corrigan JM Donaldson MS eds. Institute of medicine committee on quality of health care in a. In: To err is human: Building a safer health system. Washington (DC: National Academies Press (US. Copyright 2000 by the National Academy of Sciences. All rights reserved. 25077248

[2] Donaldson L Philip P. Patient safety: a global priority. Bull World Health Organ (2004) 82(12):892. 15654400 PMC2623103

[3] Rodziewicz TL Houseman B Hipskind JE. Medical error reduction and prevention. StatPearls. Treasure Island (FL: StatPearls Publishing (2021). Copyright © 2021, StatPearls Publishing LLC. 29763131

[4] Van Wert MJ Gandhi S Gupta I Singh A Eid SM Haroon Burhanullah M. Healthcare worker mental health after the initial peak of the COVID-19 pandemic: a US medical center cross-sectional survey. J Gen Intern Med (2022) 37(5):1169–76. doi: 10.1007/s11606-021-07251-0. PMC8734540 34993856

[5] Hughes RG ed. Advances in patient safety. In: Patient safety and quality: An evidence-based handbook for nurses. Rockville (MD: Agency for Healthcare Research and Quality (US. 21328752

[6] Hodkinson A Tyler N Ashcroft DM Keers RN Khan K Phipps D. Preventable medication harm across health care settings: a systematic review and meta-analysis. BMC Med (2020) 18(1):313. doi: 10.1186/s12916-020-01774-9 33153451 PMC7646069

[7] Nalesso F Garzotto F Rossi B Cattarin L Simioni F Carretta G. Proactive approach for the patient safety in the extracorporeal blood purification treatments in nephrology. G Ital Nefrol (2018) 35(4):42–9. 30035442

[8] Panagioti M Khan K Keers RN Abuzour A Phipps D Kontopantelis E. Prevalence, severity, and nature of preventable patient harm across medical care settings: systematic review and meta-analysis. Bmj (2019) 366:l4185. doi: 10.1136/bmj.l4185 31315828 PMC6939648

[9] Lawati MHA Dennis S Short SD Abdulhadi NN. Patient safety and safety culture in primary health care: a systematic review. BMC Fam Pract (2018) 19(1):104. doi: 10.1186/s12875-018-0793-7 29960590 PMC6026504

[10] Alotaibi YK Federico F. The impact of health information technology on patient safety. Saudi Med J (2017) 38(12):1173–80. doi: 10.15537/smj.2017.12.20631 PMC5787626 29209664

[11] Belmont E Waller AA. The role of information technology in reducing medical errors. J Health Law (2003) 36(4):615–25. 15068277

[12] Gramaticopolo S Chronopoulos A Piccinni P Nalesso F Brendolan A Zanella M. Extracorporeal CO2 removal–a way to achieve ultraprotective mechanical ventilation and lung support: the missing piece of multiple organ support therapy. Contrib Nephrol (2010) 165:174–84. doi: 10.1159/000313757 20427968

[13] Vaara ST Bellomo R. Extra-renal indications for continuous renal replacement therapy. Contrib Nephrol (2018) 194:90–8. doi: 10.1159/000485605 29597220

[14] Nalesso F Garzotto F Cattarin L Innico G Gobbi L Calò LA. Impact of different hemodiafiltration solutions on ionemia in long-term CRRT. Int J Artif Organs (2021) 44(11):807–15. doi: 10.1177/03913988211043203 34472996

[15] Husain-Syed F Ricci Z Brodie D Vincent JL Ranieri VM Slutsky AS. Extracorporeal organ support (ECOS) in critical illness and acute kidney injury: from native to artificial organ crosstalk. Intensive Care Med (2018) 44(9):1447–59. doi: 10.1007/s00134-018-5329-z 30043276

[16] Ronco C Ricci Z Husain-Syed F. From multiple organ support therapy to extracorporeal organ support in critically ill patients. Blood Purif (2019) 48(2):99–105. doi: 10.1159/000490694 31030203

[17] Neri M Villa G Garzotto F Bagshaw S Bellomo R Cerda J. Nomenclature for renal replacement therapy in acute kidney injury: basic principles. Crit Care (2016) 20(1):318. doi: 10.1186/s13054-016-1489-9 27719682 PMC5056503

[18] Villa G Neri M Bellomo R Cerda J De Gaudio AR De Rosa S. Nomenclature for renal replacement therapy and blood purification techniques in critically ill patients: practical applications. Crit Care (2016) 20(1):283. doi: 10.1186/s13054-016-1456-5 27719676 PMC5056485

[19] Nalesso F Garzotto F. Nomenclature for renal replacement therapies in chronic patients. Nephrol Dial Transpl (2020) 35(6):933–6. doi: 10.1093/ndt/gfaa009 32040171

[20] Nalesso F Cattarin L Calo LA Garzotto F. The dialyzer identification code (DIC): A filter characteristics codification for dialyzer choice in renal replacement therapy. Artif Organs (2020) 44(11):1220–23. doi: 10.1111/aor.13738 32441824

[21] Krautscheid LC. Improving communication among healthcare providers: preparing student nurses for practice. Int J Nurs Educ Scholarsh (2008) 5:Article40. 18976237 10.2202/1548-923X.1647

[22] Hwang JI Kim SW. Using an early warning score for nurse shift patient handover: Before-and-after study. Asian Nurs Res (Korean Soc Nurs Sci) (2021) 16(1):18–24. doi: 10.1016/j.anr.2021.12.005 34974179

[23] Galatzan Benjamin J Carrington Jane M. Communicating data, information, and knowledge in the nursing hand-off. Comput Inform Nurs (2022) 40(1):21–7. doi: 10.1097/CIN.0000000000000806 34347647

[24] Kenaga H Markova T Stansfield RB McCready T Kumar S. Using a direct observation tool (TOC-CEX) to standardize transitions of care by residents at a community hospital. Ochsner J (2021) 21(4):381–6. doi: 10.31486/toj.20.0154 PMC8675625 34984053

[25] Salik I Ashurst JV. Closed loop communication training in medical simulation. StatPearls. Treasure Island (FL: StatPearls Publishing (2021). Copyright © 2021, StatPearls Publishing LLC. 31751089

[26] Whittingham KA Oldroyd LE. Using an SBAR - keeping it real! demonstrating how improving safe care delivery has been incorporated into a top-up degree programme. Nurse Educ Today (2014) 34(6):e47–52. doi: 10.1016/j.nedt.2013.11.001 24559799

[27] Thomas CM Bertram E Johnson D. The SBAR communication technique: teaching nursing students professional communication skills. Nurse Educ (2009) 34(4):176–80. doi: 10.1097/NNE.0b013e3181aaba54 19574858

[28] De Meester K Verspuy M Monsieurs KG Van Bogaert P. SBAR improves nurse-physician communication and reduces unexpected death: a pre and post intervention study. Resuscitation (2013) 84(9):1192–6. doi: 10.1016/j.resuscitation.2013.03.016 23537699

[29] McGowan J Wojahn A Nicolini JR. Risk management event evaluation and responsibilities. StatPearls. Treasure Island (FL: StatPearls Publishing (2022). Copyright © 2022, StatPearls Publishing LLC. 32644752

[30] Haugen AS Sevdalis N Søfteland E. Impact of the world health organization surgical safety checklist on patient safety. Anesthesiology (2019) 131(2):420–5. doi: 10.1097/ALN.0000000000002674 31090552

[31] Thomassen Ø Storesund A Søfteland E Brattebø G. The effects of safety checklists in medicine: a systematic review. Acta Anaesthesiol Scand (2014) 58(1):5–18. doi: 10.1111/aas.12207 24116973

[32] Weaver SJ Dy SM Rosen MA. Team-training in healthcare: a narrative synthesis of the literature. BMJ Qual Saf (2014) 23(5):359–72. doi: 10.1136/bmjqs-2013-001848 PMC3995248 24501181

[33] Edwards MT. An assessment of the impact of just culture on quality and safety in US hospitals. Am J Med Qual (2018) 33(5):502–8. doi: 10.1177/1062860618768057 29658295

[34] Thomas EJ Sexton JB Neilands TB Frankel A Helmreich RL. The effect of executive walk rounds on nurse safety climate attitudes: a randomized trial of clinical units[ISRCTN85147255] [corrected]. BMC Health Serv Res (2005) 5(1):28. 15823204 10.1186/1472-6963-5-28 PMC1097728

[35] La Pietra L Calligaris L Molendini L Quattrin R Brusaferro S. Medical errors and clinical risk management: state of the art. Acta Otorhinolaryngol Ital (2005) 25(6):339–46. PMC2639900 16749601

[36] Ronco C Bagshaw SM Bellomo R Clark WR Husain-Syed F Kellum JA. Extracorporeal blood purification and organ support in the critically ill patient during COVID-19 pandemic: Expert review and recommendation. Blood Purif (2021) 50(1):17–27. doi: 10.1159/000508125 32454500 PMC7270067

[37] Ronco C Navalesi P Vincent JL. Coronavirus epidemic: preparing for extracorporeal organ support in intensive care. Lancet Respir Med (2020) 8(3):240–1. doi: 10.1016/S2213-2600(20)30060-6 PMC7154507 32035509

[38] Neyra JA Connor MJ Jr Tolwani A. Preparedness of kidney replacement therapy in the critically ill during COVID-19 surge. Kidney Int Rep (2020) 5(7):961–4. doi: 10.1016/j.ekir.2020.05.029 PMC7275991 32642604

[39] Garzotto F Comoretto RI Ostermann M Nalesso F Gregori D Bonavina MG. Preventing infectious diseases in intensive care unit by medical devices remote control: Lessons from COVID-19. J Crit Care (2021) 61:119–24. doi: 10.1016/j.jcrc.2020.10.014 PMC7588313 33157307

[40] De Micco F De Benedictis A Fineschi V Frati P Ciccozzi M Pecchia L. From syndemic lesson after COVID-19 pandemic to a "Systemic clinical risk management" proposal in the perspective of the ethics of job well done. Int J Environ Res Public Health (2021) 19(1):15. doi: 10.3390/ijerph19010015 35010289 PMC8750949

[41] Atiomo W Weir P Kean L. Impact of new clinical policies during the COVID-19 pandemic on clinical incidents and complaints at a UK teaching hospital. Int J Environ Res Public Health (2021) 18(8):3979. doi: 10.3390/ijerph18083979 33918909 PMC8070505

[42] Ryynänen S Vasari P. Restoring the performance of a health care organization following the first wave of COVID-19 by using patient complaint data. J Patient Exp (2021) 8:2374373521996267. doi: 10.1177/2374373521996267 34179369 PMC8205405

[43] Tambone V Boudreau D Ciccozzi M Sanders K Campanozzi LL Wathuta J. Ethical criteria for the admission and management of patients in the ICU under conditions of limited medical resources: A shared international proposal in view of the COVID-19 pandemic. Front Public Health (2020) 8:284. doi: 10.3389/fpubh.2020.00284 32612972 PMC7308475

[44] Foti F De-Giorgio F Vetrugno G. Communication and resolution programs in the COVID-19 era: A unique opportunity to enhance patient safety (and save money). J Patient Saf (2021) 17(3):174. doi: 10.1097/PTS.0000000000000835 33734206

[45] Adelman JS Gandhi TK. COVID-19 and patient safety: Time to tap into our investment in high reliability. J Patient Saf (2021) 17(4):331–3. doi: 10.1097/PTS.0000000000000843 33797461

[46] Lodi CA Vasta A Hegbrant MA Bosch JP Paolini F Garzotto F. Multidisciplinary evaluation for severity of hazards applied to hemodialysis devices: an original risk analysis method. Clin J Am Soc Nephrol (2010) 5(11):2004–17. doi: 10.2215/CJN.01740210 PMC3001776 20813858

[47] Garzotto F Ceresola E Panagiotakopoulou S Spina G Menotto F Benozzi M. COVID-19: ensuring our medical equipment can meet the challenge. Expert Rev Med Devices (2020) 17(6):483–9. doi: 10.1080/17434440.2020.1772757 32434400

[48] Nalesso F Garzotto F Cattarin L Gobbi L Qassim L Sgarabotto L. A continuous renal replacement therapy protocol for patients with acute kidney injury in intensive care unit with COVID-19. J Clin Med (2020) 9(5):1529. doi: 10.3390/jcm9051529 32438617 PMC7291081

[49] Nalesso F Cattarin L Calò LA. Regional citrate anticoagulation dose for continuous renal replacement therapy. Nephrol (Carlton) (2020) 25(4):361. doi: 10.1111/nep.13671 31642131

[50] Stevens JS Velez JCQ Mohan S. Continuous renal replacement therapy and the COVID pandemic. Semin Dial (2021) 34(6):561–6. doi: 10.1111/sdi.12962 PMC8242500 33705575

